# Transovarial Transmission of *Orientia tsutsugamushi* in *Leptotrombidium palpale* (Acari: Trombiculidae)

**DOI:** 10.1371/journal.pone.0088453

**Published:** 2014-04-10

**Authors:** Eun Hee Shin, Jong Yul Roh, Won Il Park, Bong Gu Song, Kyu-Sik Chang, Wook-Gyo Lee, Hee Il Lee, Chan Park, Mi-Yeoun Park, E-Hyun Shin

**Affiliations:** 1 Division of Medical Entomology, Center for Immunology and Pathology, Korea National Institute of Health, Cheongwon, South Korea; 2 Division of Biosafety Evaluation and Control, Korea National Institute of Health, Cheongwon, South Korea; 3 Division of Arboviruses, Center for Immunology and Pathology, Korea National Institute of Health, Cheongwon, South Korea; University of Texas Medical Branch, United States of America

## Abstract

Transovarial transmission of *Orientia tsutsugamushi* in colonies of *Leptotrombidium palpale* was studied in the parent and F_1_ and F2 generations. Both transovarial transmission and filial infection rates were 100% in the parent and F_1_ generations of *Leptotrombidium palpale*. The filial infection rate in the F_1_ generation was 100%, but it declined to 94.3% in the F_2_ progeny. The sex ratio of the F_1_ generation from infected *L. palpale* was 1∶0.8 (male:female) and the proportion of males was relatively high. This study is the first to report on the transovarial transmission of *O. tsutsugamushi* in *L. palpale*. High transovarial transmission rates in *L. palpale* suggest that this species might be one of the major vectors of tsutsugamushi disease in Korea.

## Introduction

Scrub typhus, better known as tsutsugamushi disease, is an acute and febrile disease caused by *Orientia tsutsugamushi* infection. This disease, which is transmitted by the bite of infected chiggers was first reported in Korea in 1951 [7]. The incidence of scrub typhus has increased remarkably in Korea. A total of 8,604 cases of scrub typhus were reported in 2012 (http://stat.cdc.go.kr). Seven species—*Euschoengastia koreaensis, Leptotrombidium orientale, L. scutellare, L. pallidum, L. palpale, L. zetum and Neotrombicula japonica*—are considered to be the major vector species in Korea [6], [12], [13], [14].

Because the larval stage is the only parasitic stage of *O. tsutsugamushi*, to maintain disease transmission, it is necessary for *O. tsutsugamush* to be transmitted transstadially through the nymph and adult stages and transovarially transmitted through the eggs to the progenies [2]. The efficiency of transmission of *Orientia* by infected chiggers is important in determining how the disease is maintained in nature. Previous studies on transovarial transmission occurred in *L. pallidum* [16] and *L. scutellare* [1] in Japan. Additionally, it was proven that *O. tsutsugamushi* was transmitted transovarially through eggs in the infected colonies of *L. fletcheri*, *L. arenicola*, *L. deliense*, *L. imphalum*, and *L. chiangraiensis* [3], [8], [11], [15].

It is known that males are aberrant hosts of *O. tsutsugamushi* [18], and infected males have been recorded previously in only two species, *L. pallidum* [16] and *L. imphalum* [8], [9]. We studied the transovarial transmission of *O. tsutsugamushi* in two generations (Parent and F_1_) of naturally-infected *L. palpale* colonies. Two parameters, transovarial transmission rate and filial infection rate, were studied. Oviposition and hatching rates in naturally infected and uninfected *L. palpale* were also compared.

## Materials and Methods

### Collection of chiggers

The animal protocol used in this study was reviewed and approved based on ethical procedures and scientific care by the KCDC-Institutional Animal Care and Use Committee (KCDC-IACUC; KCDC-12-032-1A). Engorged larval chiggers were collected from wild rodents, which were captured in March 2010 from Jangan-myeon, Hwaseong-si Gyeonggi Province, Korea. There was no need for specific permission for using these collecting sites, because these sites were not located at national parks or protected areas and installation of traps was supported by Public Health Center in Hwaseong-si. A total of 50 Sherman live folding traps (3×3×9 inch), baited with a peanut butter spread paper, set up at five points in the collection site with 5 m intervals and collected at next day morning. A total of 20 wild rodents were captured. The captured wild rodents were transferred individually into small cages made of stainless steel and each cage was placed on a petri dish containing water. The fully engorged larvae were collected from the water surface every day. These parent and F_1_ generations of trombiculid mites were used in this study.

### Rearing of chiggers under laboratory conditions

The collected engorged larvae were reared in plastic containers (50 mm diameter, 40 mm height) containing plaster of calcium sulfate hemihydrate with charcoal powder (9∶1) to maintain the humidity level in the incubator. Deutonymphs and adults were fed with the eggs of Collembola (*Sinella curviseta*). When chiggers developed into adults, their sexuality was determined by observing the presence of genital setae located in the genital pore by using a stereomicroscope [20]. Males and females were maintained in rearing containers. When the spermatophore in males was observed, the females were placed into the rearing containers for mating. Egg-laying female mites were observed daily. The males were then removed from the rearing container prevent cannibalization of the eggs. After the eggs hatched, the larvae were attached on the ears of mice for feeding.

### Detection of *O. tsutsugamushi* in chiggers

DNA was extracted from chigger mites using the G-spin total DNA extraction kit (iNtRON Biotechnology, Korea). The 56-kDa genes of *O. tsutsugamushi* were detected using a nested PCR assay performed as described in [4]. Primers 34 (5′-TCA AGC TTA TTG CTA GTG CAA TGT CTGC-3′) and 55 (5′-AGG GAT CCC TGC TGC TGT GCT TGC TGC G-3′) were used for the first PCR, and second PCR primers 10 (5′-GAT CAA GCT TCC TCA GCC TAC TAT AAT GCC-3′) and 11 (5′-CTA GGG ATC CCG ACA GAT GCA CTA TTA GGC-3′) were used to amplify a 483-bp fragment. In the first PCR, 5 µL of template DNA of chigger mites was added to the PCR premix (Bioneer, Korea). The cycling conditions used were as follows: 94°C for 5 min followed by 30 cycles of 94°C for 30 sec, 60°C for 2 min, and 72°C for 2 min, and a final extension of 72°C for 10 min. For the second PCR, 2 µL of the first PCR product was amplified by the same procedure as described above, except the use of the second PCR primer pairs as follows. The second PCR products were analyzed by electrophoresis on a 1.5% agarose gel. The second PCR products size was 483 bp. The nucleotide sequence of the nested PCR products was analyzed using the BLAST program of NCBI (http://blast ncbi.nlm. nih.gov) to confirm whether the gene is from *O. tsutsugamushi*.

## Results

We collected 1,138 engorged larvae from twenty wild rodents, *Apodemus agrarius* Thomas were captured in Jangan-myeon, Hwaseong-si (Table 1). Two *O. tsutsugamushi*-infected female mites were collected from two wild rodents (A1, A2). The positive female mites (P1 and P2) produced 25 and 24 eggs and 18 and 16 larvae were hatched from these, respectively. The transovarial infection rate in *L. palpale* is summarized in Table 2. Transovarial infection rates in *L. palpale* parents (2/2) and F_1_ adults (8/8) were 100%. The filial infection rate in the F_1_ generation was 100% (34/34), but this rate slightly declined to 94.3% in the F_2_ larvae (160/169).

**Table 1:** The chigger mite collected from the captured wild rodents in Hwaseong.

Locallity	Coll. Date	Host		No. of chiggers
		No. Host	Species	
Hwaseong	17/Mar/10	A1	*A. agrarius*	100
		A2	*A. agrarius*	95
		A3	*A. agrarius*	46
		A4	*A. agrarius*	31
		A5	*A. agrarius*	111
		A6	*A. agrarius*	42
		A7	*A. agrarius*	35
		A8	*A. agrarius*	19
		A9	*A. agrarius*	40
		A10	*A. agrarius*	36
		A11	*A. agrarius*	25
		A12	*A. agrarius*	30
		A13	*A. agrarius*	55
		A14	*A. agrarius*	64
		A15	*A. agrarius*	89
		A16	*A. agrarius*	79
		A17	*A. agrarius*	108
		A18	*A. agrarius*	55
		A19	*A. agrarius*	40
		A20	*A. agrarius*	35
Total				1,138
Average (Chigger Index)				56.9

**Table 2:** *Orientia tsutsugamushi* infection rates in F_1_ and F_2_ generations of *Leptotrombidium palpale*.

Wild rodent	Parent	F1						F2
		eggs	Larva	Infection rate (%) (No. of chiggers tested)			Adult	Infection rate (%) (No. of chiggers tested)
				Larva to Nymph	Male	Female		
A* 1	P1	25	18	100(8)	100(4)	100(6)	F1A1	100(25)
							F1A2	95.0(20)
							F1A3	93.3(15)
							F1A4	100(17)
							F1A5	88.2(17)
							F1A6	96.8(31)
					Subtotal			96.0(125)
A* 2	P2	24	16	100(7)	100(6)	100(2)	F1A7	87.5(24)
							F1A8	95.0(20)
					Subtotal			90.9(44)
Mean infection rate (No. of total tested)				100(15)	100(10)	100(8)		94.3(169)

A^*^: *Apodemus agrarius*.

Both infected and uninfected *L. palpale* females produced eggs for 16 weeks. Infected females laid 32.6±6.7 eggs per female and uninfected ones laid 31.5±7.7 eggs per female. The hatching rate of the eggs from infected females was 64.8±14.4% and that in uninfected females was 74.5±8.3%. The number of eggs from infected and uninfected females were not significantly different (P>0.05), while the hatching rate in the infected cohort was lower than that in the uninfected cohort (P<0.05) (Table 3).

**Table 3:** Fecundity and hatching rate of *Leptotrombidium palpale*.

Infected/Uninfected (n)	Egg (Mean±SD)	Chigger (Mean±SD)	Rate of hatching (%) (Mean±SD)
Infected (8)	32.6±6.7^NS^	21.1±5.3^NS^	64.8±14.4^a^
Uninfected (8)	31.5±7.7^NS^	23.3±5.6^NS^	74.5±8.3^b^

NS : Not significant.

a–b : Values within a column with different superscripts are significant at P<0.05.

## Discussion

Successful transovarial transmission in chiggers is important in the epidemiology of scrub typhus [8]. Previous studies on transovarial transmission was conducted in *L. pallidum* [16] and *L. scutellare* [1] vector species in Korea. This is the first report on transovarial transmission of *L. palpale*, which is a vector species of scrub typhus in Korea.

Transovarial and filial infection rates in *L. palpale* are similar to those in *L. pallidum* [17]. Transovarial and fillial infection rates in *L. pallidum* were 100% [19] in the F_1_ generation, but these rates declined to 97% in the F_2_ and 90% in the F_3_. The fillial infection rate rapidly decreased in the succeeding generations. Electron microscopic observations revealed that *O. tsutsugamushi* did not always invade the oocytes in the ovaries of infected females [5]. Phasomkusolsil et al. [8] recorded that the filial infection rate in F_1_ of *L. imphalum* was 100%, which declined to 62.3% in the F_2_.

Infected male chiggers were reported in *L. fletcheri* [10], [11], *L. arenicola* [10], [15], *L. pallidum* [16], and *L. imphalum* [8], [9]. However, the occurrence of infected males was rare in *L. fletcheri* (1∶107.8), *L. arenicola* (1∶905), and *L. imphalum* (1∶64.5). In our study, the male to female sex ratio in *L. palpale* F_1_ generation was 1∶0.8. This result is similar to the sex ratio (1∶1.09) in *L. pallidum* [16].

In this study, *L. palpale* females laid eggs for 16 weeks, but it was for 28 weeks and longer in *L. imphalum* [10]. This period might vary with the species and rearing conditions.

To date, seven species—*Euschoengastia koreaensis, L. orientale, L. scutellare, L. pallidum, L. palpale, L. zetum, and Neotrombicula japonica*—have been considered as vectors in Korea [6], [12], [13], [14]. In order to determine the vector species of tsutsugamushi disease, chiggers collected from wild rodents were tested for *O. tsutsugamushi* infection using an indirect immunofluorescent antibody (IFA) test and polymerase chain reaction (PCR) methods. In this case, chiggers were a possibly temporarily infected through feeding on the fluids of infected wild rodents. In order to determine the vector species more clearly, it is important to investigate whether the unfed larvae collected from the soil were infected by the pathogens or not, or their transovarial transmission should be confirmed through successive rearing.

This is the first study describing transovarial transmission of *O. tsutsugamushi* in *L. palpale* in Korea. Further studies are needed to confirm transovarial transmission of other species for vector determination and to investigate the distribution of tsutsugamushi disease associated with the vector species.
